# Supplementary material for: Complexome profiling of the Chlamydomonas psb28 mutant reveals TEF5 as an early PSII assembly factor
Source: Plant Cell. 2025 Mar 17;37(6):koaf055. doi: 10.1093/plcell/koaf055 (PMC12164590; doi:10.1093/plcell/koaf055)
Supplement: koaf055_Supplementary_Data [file koaf055_supplementary_data.zip › psb28_interactive.zip!/plots/intensity/qualityPlots/peptides/CYN19-2_Cre01.g002300.t1.2.html]

### Cre01.g002300.t1.2

Trivial name: CYN19-2  
SeqCoverage [median]: 10.50  
Mass: 18.38 kDa  
  
 

| Sequence | MissedCleavage | Mass [Da] | wtIntensity | muIntensity | Score |
| --- | --- | --- | --- | --- | --- |
| IEMELYADDVPK | 0 | 1421.67 | 0.00 | 1012.91 | 102.06 |
|  |  |  | 0.0 | 1012.9 |  |
